# Novel Robotic-Assisted Cryobiopsy for Peripheral Pulmonary Lesions

**DOI:** 10.1007/s00408-022-00578-3

**Published:** 2022-10-10

**Authors:** Catherine L. Oberg, Ryan P. Lau, Erik E. Folch, Tao He, Reza Ronaghi, Irawan Susanto, Colleen Channick, Rodrigo Garcia Tome, Scott Oh

**Affiliations:** 1 Section of Interventional Pulmonology, David Geffen School of Medicine at UCLA, Los Angeles, CA 90095 USA; 2 Department of Pathology and Laboratory Medicine, David Geffen School of Medicine at UCLA, Los Angeles, CA 90095 USA; 3 Section of Interventional Pulmonology, Massachusetts General Hospital, Harvard Medical School, Boston, MA 02210 USA; 4 Department of Medicine, Division of Pulmonary and Critical Care Medicine, David Geffen School of Medicine at UCLA, 10833 Le Conte Avenue, Los Angeles, CA 90095 USA

**Keywords:** Robotic bronchoscopy, Lung nodule, Cryobiopsy, Lung cancer, Advanced bronchoscopy, Interventional pulmonology

## Abstract

**Purpose:**

Tissue acquisition in lung cancer is vital for multiple reasons. Primary reasons reported for molecular testing failure in lung cancer biopsy specimens include insufficient amount of tumor cells provided and inadequate tissue quality. Robotic bronchoscopy is a new tool enabling peripheral pulmonary lesion sampling; however, diagnostic yield remains imperfect possibly due to the location of nodules adjacent to or outside of the airway. The 1.1-mm cryoprobe is a novel diagnostic tool and accesses tissue in a 360-degree manner, thus potentially sampling eccentric/adjacent lesions. This study examines the diagnostic yield of the cryoprobe compared to standard needle aspiration and forceps biopsy. It additionally evaluates yield for molecular markers in cases of lung cancer.

**Methods:**

This is a retrospective analysis of 112 patients with 120 peripheral pulmonary lesions biopsied via robotic bronchoscopy using needle aspirate, forceps, and cryobiopsy.

**Results:**

The overall diagnostic yield was 90%. Nearly 18% of diagnoses were made exclusively from the cryobiopsy sample. Molecular analysis was adequate on all cryobiopsy samples sent. Digital imaging software confirmed an increase in quantity and quality of samples taken via cryobiopsy compared to needle aspirate and traditional forceps biopsy.

**Conclusion:**

Using the 1.1-mm cryoprobe to biopsy PPN combined with the Ion robotic bronchoscopy system is safe, feasible, and provides more diagnostic tissue than needle aspirates or traditional forceps biopsies. The combination of cryobiopsy with robotic-assisted bronchoscopy increased diagnostic yield, likely due to its 360-degree tissue acquisition which is beneficial when targeting extraluminal lesions adjacent to the airway.

## Introduction

Tissue acquisition in lung cancer is critical for diagnosis, histologic subtyping, and identifying driver mutations [1]. Despite significant improvements in response rates using novel targeted therapies, many patients do not receive available agents for various reasons. In a survey published in 2020 distributed to the International Association for the Study of Lung Cancer (IASLC) members, the primary reasons reported for molecular testing failure were insufficient amount of tumor cells provided and inadequate tissue quality [2].

Robotic-assisted bronchoscopy is the newest technology available to biopsy peripheral pulmonary nodules (PPN). The Ion robotic system (Intuitive Surgical, Sunnyvale, CA) utilizes proprietary shape-sensing technology for navigation to PPN, including those which are extraluminal and/or abutting the pleural surface [3]. While a recent study reported lesion localization of 97% [4], diagnostic yields remain lower at 83% [5]. This is likely multifactorial; however, it suggests that despite reaching the lesion with the bronchoscope, biopsy tool strategies are ineffective at acquiring targeted tissue, especially with extraluminal lesions adjacent to the airway [6].

Prior studies have reported sufficient cytologic evaluation from needle aspirate specimens taken from peripheral pulmonary nodules, although adequacy for molecular genetic testing and sensitivity for malignancy remain unsatisfactory at times. In a retrospective study evaluating 113 PeriView FLEX needle specimens, the sensitivity for malignancy was 70% and only 69% of malignant samples were suitable for molecular testing [7]. Other studies have evaluated diagnostic yield and specimen adequacy from bronchial brushing specimens obtained by radial endobronchial ultrasound (REBUS) guidance in peripheral pulmonary lesions. In 71 patients, the overall diagnostic yield was only 71%; however, in the 30 patients with adenocarcinoma, 100% of samples were viable for further molecular testing. [8]

A novel tool for lung biopsy, cryoprobes are insulated catheters inserted through a bronchoscope and can extend to the lung periphery. The distal metal tip cools to −79 °C within seconds via the Joule–Thomson effect [9]. Transbronchial lung cryobiopsies (TBLCB) are used in patients with interstitial lung disease (ILD) in order to acquire larger tissue samples and prevent crush artifact that can occur with forceps biopsies [10]. Despite freezing, tissue architecture and cytologic findings are preserved, including nuclear details, such as mitotic activity and nuclear-cytoplasmic ratio. Additionally, the TBLCB obtain tissue in a 360-degree fashion; whereas, traditional tools only biopsy in one plane. Therefore, an eccentric PPN may be missed if the opposite side of the airway is biopsied; whereas, a 360-degree biopsy will sample tissue from all sides. In a recent study sampling PPN using a 1.9-mm (mm) cryoprobe through a conventional bronchoscope, the diagnostic yield was increased by 8.6% exclusively based on the cryobiopsy [11].

In this pilot study, we evaluated the feasibility and impact on tissue acquisition of the 1.1-mm cryoprobe (Erbe, Tuebingen, Germany) used via the Ion robotic bronchoscopy system. To our knowledge, this is the first study of its kind.

## Methods

### Patient Selection

From October 2021 to August 2022, sequential patients with a lung nodule or mass at the University of California, Los Angeles (UCLA), who underwent robotic bronchoscopy were retrospectively identified through the Institutional Review Board-approved UCLA, Interventional Pulmonology Research Consortium (IRB#19-000779).

### Procedural Technique

**Fig. 1:**
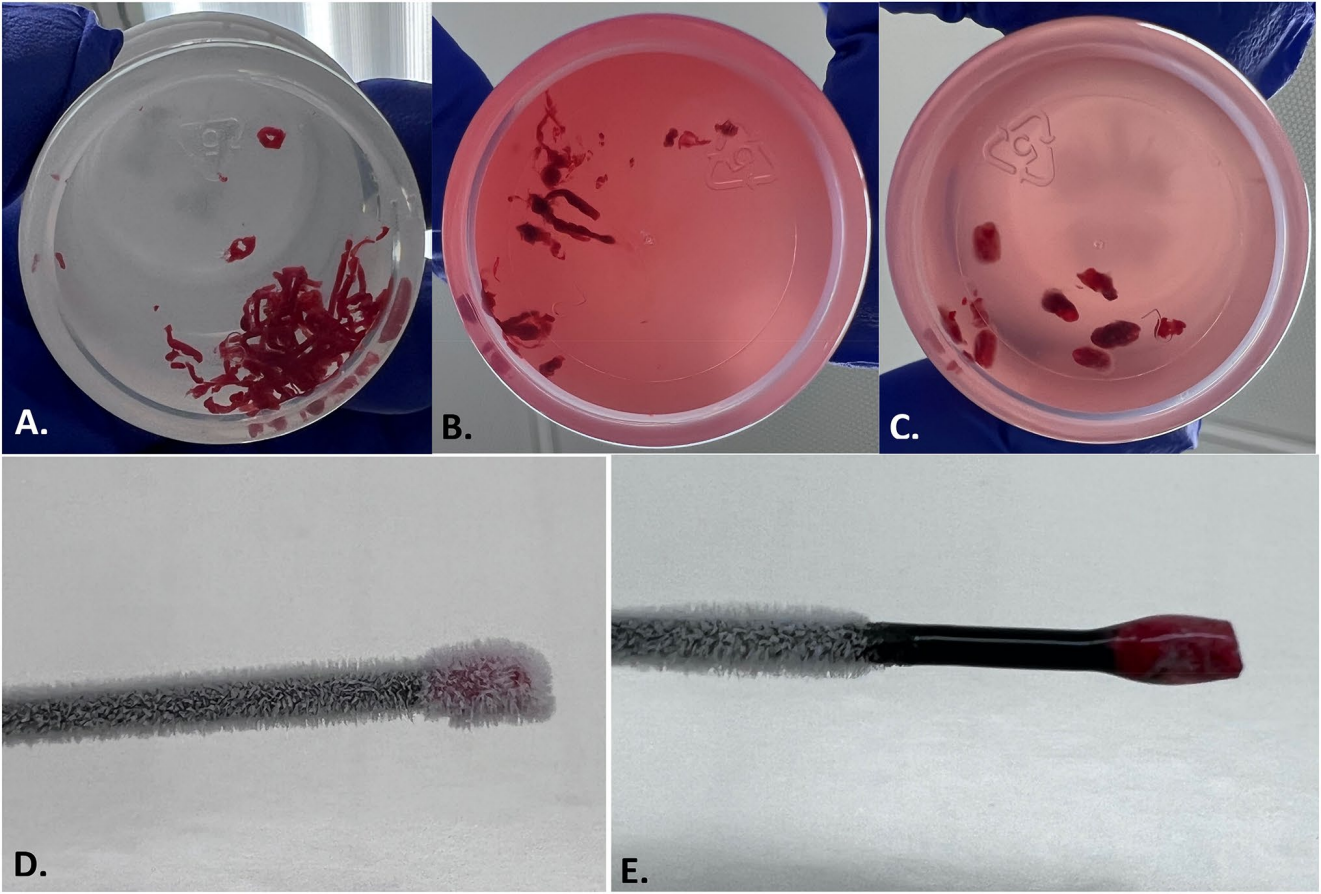
Gross comparison of biopsy specimens. **a** 21-gauge needle specimen in formalin. **b** Standard forceps biopsy specimens in formalin. **c** 1.1-mm cryobiopsy specimens in formalin. **d** Frozen cryobiopsy specimen on probe. **e** Thawed cryobiopsy specimen on probe

Every patient had a pre-procedure high-resolution computed tomography (HRCT) chest, which was used to plan the procedure as well as calculate the lesion size, location, presence or absence of bronchus sign, and distance from the pleura. All patients received general anesthesia with ventilation parameters set to optimize biopsy of the peripheral lesion [12]. The shape-sensing robotic bronchoscopic system was used exclusively to biopsy all lesions. REBUS (Olympus Medical, Japan) and conventional two-dimensional fluoroscopy were used to confirm lesion location; REBUS view was recorded as either eccentric, concentric, or negative. Biopsies were obtained in a standardized fashion. First, either a 19-gauge or 21-gauge needle (Intuitive Surgical, Sunnyvale, CA) was used with a minimum of three passes. Forceps biopsies (Endojaw, Olympus Medical, Japan) were then obtained with a minimum of five biopsies taken. Finally, the 1.1-mm cryoprobe was used to take three to four additional biopsies. The probe was tested in normal saline immediately prior to the biopsies to confirm freezing time. It was then inserted through the robotic working channel, its location confirmed via fluoroscopy, and the confirmed freezing time applied, typically 4–5 s. The sample was removed through the working channel, leaving the robotic catheter in place for mechanical hemostatic tamponade, if needed, as well as repeat biopsy without the need to renavigate. Of note, given the size of the biopsies these procedures were done through an 8.5-mm endotracheal tube and no balloon occlusion device was placed prophylactically as has been described for cryobiopsy in ILD [13]. Figure 1 shows a frozen and a thawed cryobiopsy specimen as well as a gross comparison of needle aspirate tissue, forceps biopsies, and cryobiopsies.

### Tissue Processing and Quantification

Needle aspirates, forceps transbronchial biopsies, and TBLCB were all placed in formalin. In the first 40 cases, needle aspirates were graded based on cellularity. Forceps biopsies and TBLCB were all analyzed for the presence of crush artifact, number of diagnostic fragments present, and amount of diagnostic material present in microns. In any case of lung adenocarcinoma or squamous cell carcinoma, local immunohistochemistry (IHC) and fluorescence in situ hybridization (FISH) were performed locally as well as send out NGS using a targeted 648-gene oncology panel. Slides were reviewed at the time of diagnosis for absolute tumor cellularity as well as percentage of tumor compared to normal tissue present. Pathologists identified the biopsy modality that had both a high absolute tumor cellularity and tumor percentage and that specimen was chosen for NGS.

### Statistical Methods

Standard descriptive statistics were employed using median and interquartile range for continuous variables and frequency and percentage for categorical variables.

## Results

### Patient/Lesion Characteristics

A total of 112 patients with 120 PPN were identified (Table 1). The median short and long axes dimensions of all lesions were 15.5 mm (8–25.3) and 22 mm (13–34.3), respectively. A bronchus sign was present in 48% of patients. The majority of lesions were solid at 73%, while 21% were part-solid and the remaining ground glass. Lesions were located a median of 6 mm (0–15) from the pleural surface. Fifty-four percent of patients had structural lung disease, either radiographic emphysema or ILD, conferring an increased risk of complications with lung nodule sampling.

**Table 1:** Demographics, clinical characteristics, and nodule features

Demographics and clinical characteristics	Median (IQR) or frequency (%)
	*n* = 112
Age, years	71 (64–76)
Sex	
Women	60 (54%)
Men	52 (46%)
Smoking history	66 (59%)
Pack years	25 (15–50)
Comorbidities	
Pulmonary Disease	66 (59%)
Radiographic emphysema	38 (34%)
COPD	33 (29%)
Interstitial lung disease	22 (20%)
Asthma	11 (9.8%)
Chronic respiratory failure on home oxygen	4 (3.6%)
Lung transplant	3 (2.7%)
Sarcoidosis	2 (1.8%)
Non-Pulmonary Disease	105 (94%)
Hypertension	51 (46%)
Hyperlipidemia	36 (32%)
Prior Malignancy	37 (33%)
Lung	11 (9.8%)
Breast	8 (7.1%)
Prostate	6 (5.4%)
Hematologic	6 (5.4%)
Uterine	4 (3.6%)
Renal Cell	3 (2.7%)
Melanoma	2 (5.7%)
Bladder	2 (1.8%)
Colon	2 (1.8%)
Head and Neck	2 (1.8%)
Pancreatic	1 (0.9%)
Hepatocellular	1 (0.9%)
With more than 1 malignancy	9 (8%)
Cardiac disease/stroke	23 (20.5%)
GERD	20 (17.9%)
Diabetes mellitus II	19 (17%)
Medications	
Antiplatelet agent*	20 (17.8%)
Therapeutic anticoagulation**	12 (10.7%)
Laboratory Values	
Platelet count × 10^9^ per l	249 (192.5–301)
International normalized ratio	1 (1–1.1)
Blood urea nitrogen, mg/dL	16 (13–22)
Creatinine, mg/dL	0.89 (0.74–1.1)

Nodule Features	*n* = 120
Location	
Right upper lobe	35 (29.2%)
Right middle lobe	10 (8.3%)
Right lower lobe	30 (25%)
Left upper lobe	28 (23.3%)
Lingula	3 (2.5%)
Left lower lobe	14 (11.7%)
Size	
Short axis, millimeters	15.5 (8–25.3)
Long axis, millimeters	22 (13–34.3)
Nodules 10 mm or under (long axis)	19 (15.8%)
Nodules 11–20 mm	39 (32.5%)
Nodules 21–30 mm	27 (22.5%)
Nodules > 30 mm	35 (29.2%)
Characteristics	
Solid	87 (72.5%)
Part-solid	25 (20.8%)
Ground glass	8 (6.7%)
Bronchus sign present	58 (48.3%)
Pleural distance, millimeters	6 (0–15)
*Procedure Details*	
REBUS used	120 (100%)
Concentric	67 (55.8%)
Eccentric	37 (30.8%)
No signal	16 (13.3%)

Complications	*n* = 112
Pneumothorax	6 (5.4%)
Chest tube placement	3 (2.7%)
Minor bleeding	3 (2.7%)

*Aspirin 81 mg was continued; if on clopidogrel, this was held 5 days prior to the procedure

**Any anticoagulant was held in the recommended period of time prior to the procedure

*IQR* interquartile range, *COPD* chronic obstructive pulmonary disease, *GERD* gastrointestinal reflux disease, *mg* milligrams, *dL* deciliter, *REBUS* radial endobronchial ultrasound

### Procedure Details

REBUS was used in all cases with a signal noted in 87%, 56% concentric, and 31% eccentric. The procedure was completed in all patients and none required prolonged intubation or hospitalization. Six (5.4%) patients developed a post-procedure pneumothorax; three (2.7%) required chest tube placement and were admitted for 24-h post-procedure. There was no major bleeding in any case. Major bleeding was defined as Grade 2 or higher on the Nashville Bleeding Scale [14].

### Diagnoses

A confirmed specific diagnosis was made in 101 patients and 108 nodules, indicating a per-patient diagnostic yield of 90.2%. Malignancy was confirmed in 46.7% of nodules and a benign etiology was confirmed in 43.3%. There were 17 diagnoses of fibrosis or non-specific inflammation; these cases were only considered diagnostic if follow-up imaging revealed improvement or resolution of the lesion (*n* = 10) or if a secondary biopsy modality confirmed the same result (*n* = 7). In cases with no follow-up imaging or confirmatory biopsy, these were considered false-negative results. Importantly, there were two false-negative cases that were ultimately diagnosed with a malignancy. One case was positive for squamous cell carcinoma on the linear EBUS sampling of the lymph nodes which was done at the time of the robotic bronchoscopy. The second case was a 4 × 8-mm solid lesion 17 mm from the pleura and was diagnosed as lung adenocarcinoma by CT-guided biopsy.

**Fig. 2:**
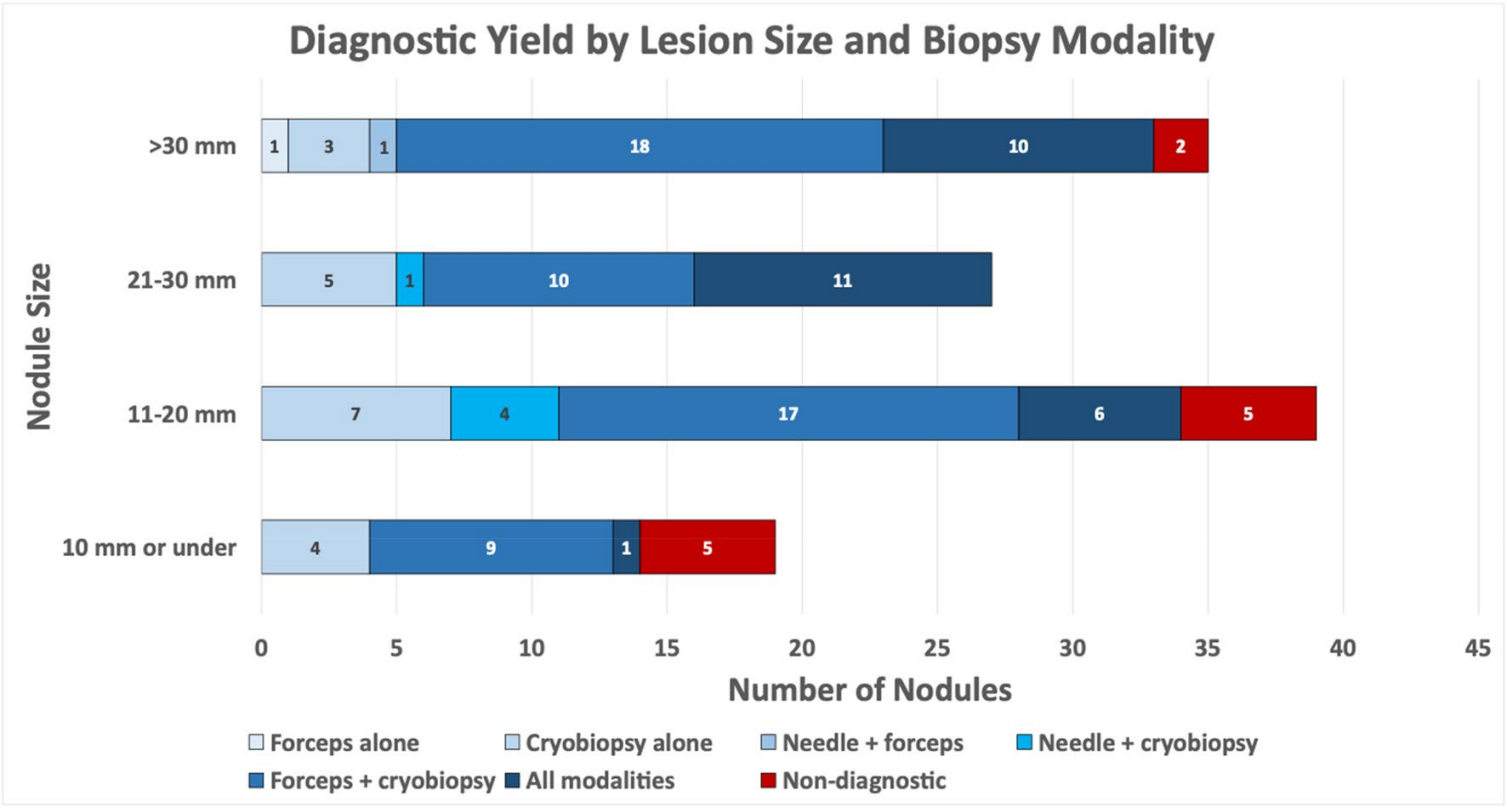
Diagnostic yield by lesion size and biopsy modality

Of the 108 diagnostic nodules, the needle sample was positive in 34 (31.5%). Transbronchial forceps biopsies provided the diagnosis in 84 cases (77.8%). Cryobiopsies were diagnostic in 105 lesions (97.2%). The forceps were the only diagnostic modality in two lesions (1.9%) and the cryobiopsies were the only diagnostic modality in 19 (17.6%). In no cases were the needle the sole diagnostic modality. See Fig. 2. Local IHC and FISH as well as NGS were performed in 49 cases of lung adenocarcinoma and squamous cell carcinoma, 29 from the cryobiopsy, 18 from the forceps biopsy, and 2 from the needle specimens. All cryobiopsy cases were adequate for both IHC/FISH and NGS; 3 forceps biopsy samples were deemed inadequate for NGS due to insufficient cellularity as well as 1 of the 2 needle samples (Table 2).

**Table 2:** Diagnoses and molecular testing

Diagnoses	Median (IQR) or Frequency (%)
	*n* = 120
Malignancy	56 (46.7%)
Adenocarcinoma, lung	34 (28.3%)
Squamous cell carcinoma, lung	15 (12.5%)
Lymphoma/lymphoproliferative disorder	3 (2.5%)
Small cell carcinoma, lung	1 (0.8%)
Renal cell carcinoma	1 (0.8%)
Endometrial	1 (0.8%)
Sarcoma	1 (0.8%)
Benign	52 (43.3%)
Granulomas	17 (14.2%)
Organizing pneumonia	9 (7.5%)
Inflammation with or without necrosis	9 (7.5%)
Fibrosis/fibroelastic scar	8 (6.7%)
Infection	7 (5.8%)
Aspergillus	3 (2.5%)
Coccidioidomycosis	3 (2.5%)
Streptococcus anginosus	1 (0.8%)
Hamartoma	2 (1.7%)
Non-Diagnostic	12 (10%)

Yield per tool, diagnostic cases	*n* = 108
Needle alone	0 (0%)
Forceps alone	2 (1.9%)
Cryobiopsy alone	19 (17.6%)
Needle + forceps	1 (0.9%)
Needle + cryobiopsy	5 (4.6%)
Forceps + cryobiopsy	53 (49.1%)
Needle + forceps + cryobiopsy	28 (25.9%)

Molecular analysis	*n* = 49
Local IHC/FISH adequacy	49 (100%)
Needle	2 (100%)
Forceps	18 (100%)
Cryobiopsy	29 (100%)
NGS adequacy	45 (81.6%)
Needle	1 (50%)
Forceps	15 (83.3%)
Cryobiopsy	29 (100%)

*IHC—immunohistochemistry, FISH—fluorescence in situ hybridization, NGS—next-generation sequencing

### Tissue Grading/Quantification

The first 40 lesion samples were pathologically characterized for cellularity, number of diagnostic fragments, presence of crush artifact, and greatest dimension of diagnostic material (Table 3). Cellularity was graded as acellular (0), paucicellular (1), moderately cellular (2), and hypercellular (3). Crush artifact was noted as none or minimal (0) or present (1). Diagnostic material was measured in microns.

**Table 3:** Pathologic quantification

Samples	Median (IQR)
	*n* = 40
Transbronchial Needle Aspirate	
Cellularity*	0 (0–1)
Diagnostic fragments**	0.5 (0–1)
Crush artifact*** (*n* = 12)	0 (0–1)
Greatest dimension diagnostic material, microns	0 (0–135.9)
Diagnostic material per fragment, microns	170.9 (0–516.3)
Transbronchial Forceps Biopsy	
Diagnostic fragments**	1 (1–3)
Crush artifact*** (*n* = 35)	1 (0–1)
Greatest dimension diagnostic material, microns	1821.5 (996–3194.7)
Diagnostic material per fragment, microns	1017.8 (795.5–1317.8)
Transbronchial Cryobiopsy	
Diagnostic fragments**	2 (1–2)
Crush artifact*** (*n* = 38)	0 (0–0.5)
Greatest dimension diagnostic material, microns	3570.6 (1974.6–4833.2)
Diagnostic material per fragment, microns	1968.2 (1195.5–3326.4)

*Cellularity: 0—Acellular, 1—Paucicellular, 2—Moderately Cellular, 3—Hypercellular

**Diagnostic Fragments: < 1—detached cellular material only

***Crush Artifact: 0—none/minimal, 1—present

Needle aspirate smears were made in 28 lesions. Eleven were acellular, 11 were paucicellular, 5 was moderately cellular, and 1 was hyper cellular. Tissue fragments from the needle were present in 23 samples; 3 had innumerable fragments, 2 had 4 fragments, 1 had 2 fragments, 9 had 1 fragment, and 8 had detached cellular material only. In the 12 nodules with quantifiable needle tissue fragments, crush artifact was identified in 4 (33.3%). A median of 170.9 microns (0–516.3) of diagnostic tissue was present per needle tissue fragment.

Forceps biopsies were evaluated in 37 cases with a median of 1 (1–3) diagnostic fragments per case. Of the 35 cases with diagnostic fragments identified, 19 (54.3%) had crush artifact present. A median of 1017.8 (795.5–1317.8) microns of diagnostic tissue was present per diagnostic fragment.

All cases had cryobiopsies performed; a median of 2 (1–2) diagnostic fragments per case was identified. In these specimens, crush artifact was present in 10 (25%). A median of 1968.2 (1195.5–3326.4) microns of diagnostic material per fragment was present.

**Fig. 3:**
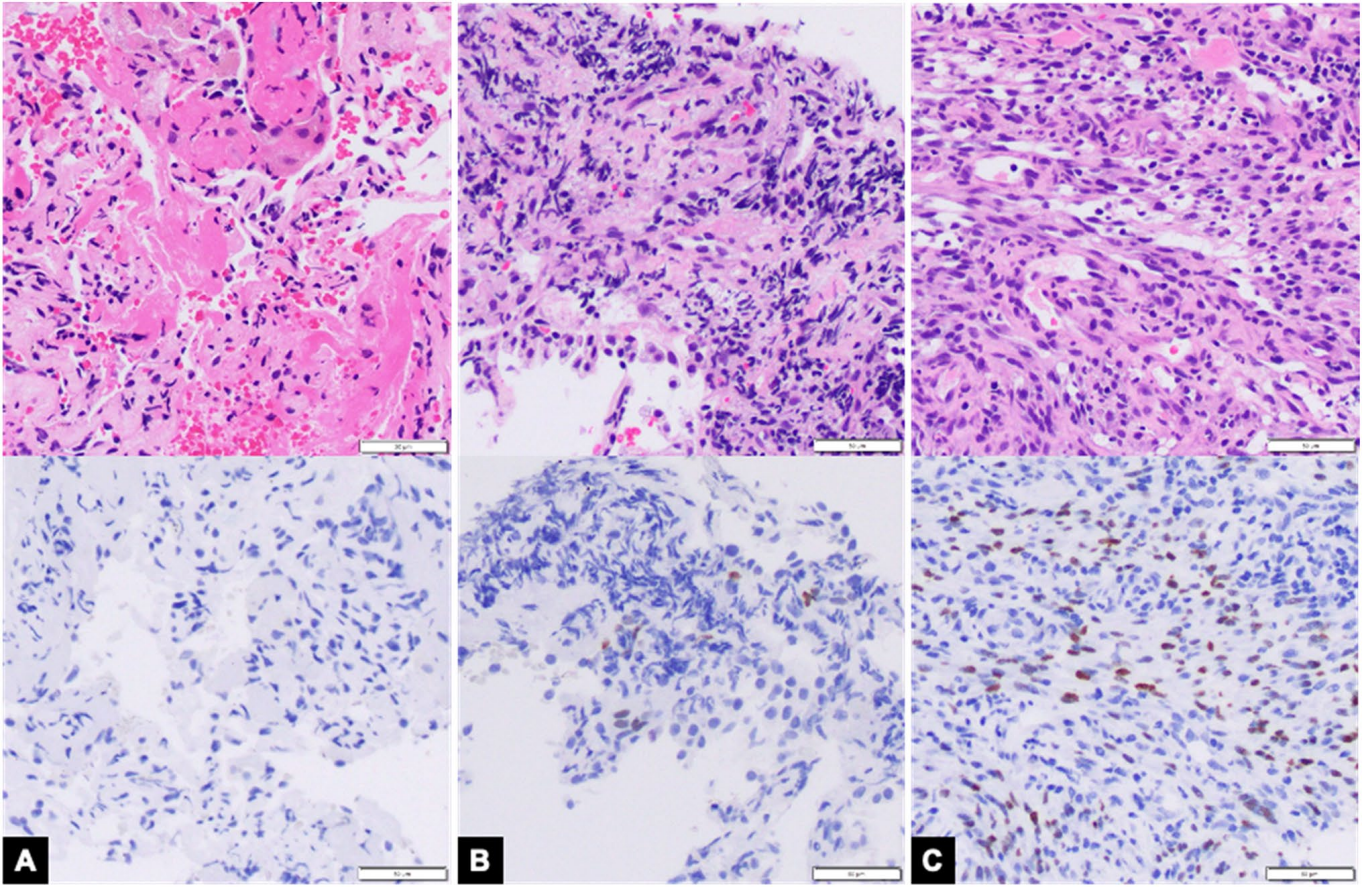
Kaposi Sarcoma. **A** Fine needle biopsy remnant tissue and blood clot showed minute unremarkable lung tissue with no diagnostic tissue (Top panel: Hematoxylin & Eosin stain, 200 × magnification; Bottom panel: HHV-8 immunohistochemistry, 200 × magnification); aspirate smears were acellular (not pictured) **B** Forceps biopsy showed one fragment of rare spindle cells with crush artifact and focal, weak HHV-8 expression (Top panel: Hematoxylin & Eosin stain, 200 × magnification; Bottom panel: HHV-8 immunohistochemistry, 200 × magnification) **C** Cryobiopsy showed Kaposi sarcoma with strong and diffuse HHV-8 expression (Top panel: Hematoxylin & Eosin stain, 200 × magnification; Bottom panel: HHV-8 immunohistochemistry, 200 × magnification)

Figure 3 shows a comparison of the needle aspirate and tissue fragment, forceps biopsy, and cryobiopsy. Fine needle aspiration biopsy showed an acellular smear; the remnant tissue and blood clot showed minute unremarkable lung tissue with no diagnostic abnormalities. The forceps biopsy showed one fragment of rare spindle cells with crush artifact and focal, weak HHV-8 expression. The cryobiopsy showed Kaposi sarcoma with strong and diffuse HHV-8 expression.

## Conclusion

This pilot study confirms the feasibility of using the 1.1-mm cryoprobe to biopsy PPN via the Ion robotic bronchoscopy system and suggests that a multimodality biopsy approach could increase diagnostic yield. All lesions were localized and tissue acquired in all cases.

Ninety percent of cases were diagnostic; notably, in nearly 18% of cases, cryobiopsy alone was the sole diagnostic modality. The needle-based samples were only positive in approximately one-third of diagnostic cases; whereas, cryobiopsies were positive in over 97%. This may be due to the 360-degree sampling provided by the cryoprobe which can access tissue from all aspects of the airway. This mechanism may be particularly helpful with lesions adjacent to the airway. The cryoprobe did not provide the diagnosis in three cases; whereas, the forceps or the needle and forceps did. The cryobiopsy specimens were grossly smaller in these cases, indicating that sampling technique such as incorrect probe placement or inadequate freezing time may have played a role. In the 49 cases of lung adenocarcinoma or squamous cell carcinoma, all cryobiopsy specimens sent were adequate for NGS; whereas, three of the forceps biopsy samples were insufficient as well as one of the two needle aspirate cell blocks sent.

The 1.1-mm cryoprobe uniformly provided larger amounts of diagnostic tissue than both the forceps biopsies and the needle aspirates. While this study was not powered to detect a statistically significant difference between groups, the amount of tissue noted in the cryoprobe samples was nearly double that of the forceps biopsies and over 10 times more than the needle samples. The tissue was also of higher quality with less crush artifact noted.

Multiple studies have shown that needle aspirates can provide adequate tissue acquisition for diagnosis and molecular analysis in lung cancer [15]. The discrepancy in our cases may be due to less high-quality material provided. Additionally, the final interpretations were not always performed by a fellowship-trained cytopathologist; therefore, the comfort level in making a diagnosis using needle aspirates may have varied.

Finally, there were no major adverse events in any patient. Six patients experienced post-procedure pneumothoraces, half of whom had radiographic emphysema or ILD. Three patients (2.7%) required chest tube placement, but none had persistent air leaks or prolonged hospital admissions. The median distance to the pleura in these six cases was 2 mm and all nodule sizes were 2 cm or less. Of note, over half of the patients in this cohort had evidence of radiographic emphysema or ILD, potentially increasing the risk of pneumothorax with biopsy. These rates are comparable to existing data in navigation bronchoscopy and are significantly lower than pneumothorax rates in percutaneous lung biopsies which approach 15% ^16, 17^. Additionally, there was no significant bleeding in any case and prophylactic balloon occlusion was not necessary. This is likely due to the smaller probe size utilized in this study compared to larger catheters used in previously described series of cryobiopsy for ILD. The robotic catheter may also have provided some measure of mechanical tamponade, increasing hemostasis. While a larger sample size is needed to definitively assess the safety of using the 1.1-mm cryoprobe via robotic bronchoscopy, this study suggests that it is safe and does not add additional risk to the procedure.

In summary, using the 1.1-mm cryoprobe to biopsy PPN combined with the Ion robotic bronchoscopy system is safe, feasible, and provides more diagnostic tissue than needle aspirates or traditional forceps biopsies. The combination of cryobiopsy with robotic-assisted bronchoscopy increased diagnostic yield, likely due to its 360-degree tissue acquisition which is beneficial when targeting extraluminal lesions adjacent to the airway. Larger studies are needed to confirm these findings.
